# Supplementary material for: Root Metabolism and Effects of Root Exudates on the Growth of Ralstonia solanacearum and Fusarium moniliforme Were Significantly Different between the Two Genotypes of Peanuts
Source: Genes (Basel). 2023 Feb 20;14(2):528. doi: 10.3390/genes14020528 (PMC9956333; doi:10.3390/genes14020528)
Supplement: Supplementary file 1 [file genes-14-00528-s001.zip › Supplemental Fig.pdf]

a

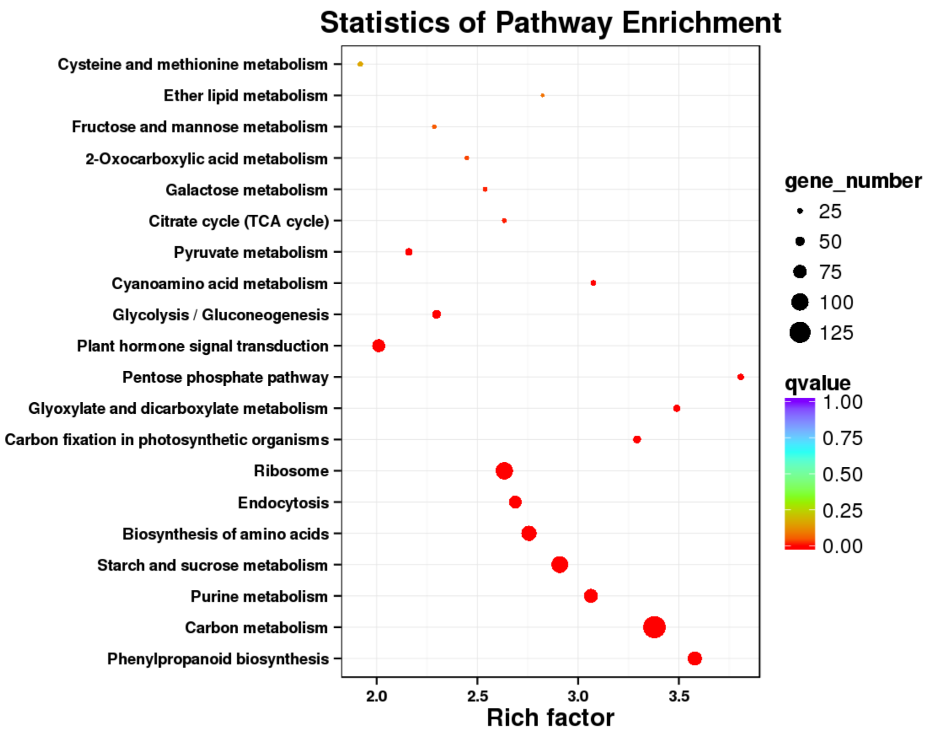

b

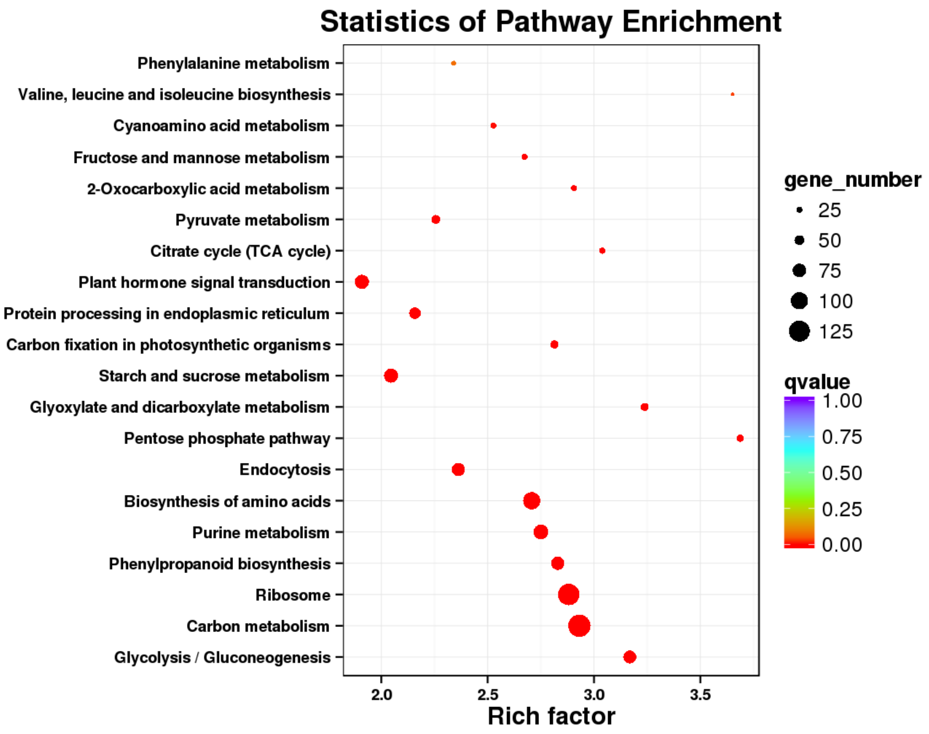

Figure S1. Enrichment analysis for KEGG pathways of DGEs between *A. correntina* and GH85. a: *A. correntina* -30d_vs_GH85-30d. b: *A. correntina* -60d _vs_GH85-60d.

a

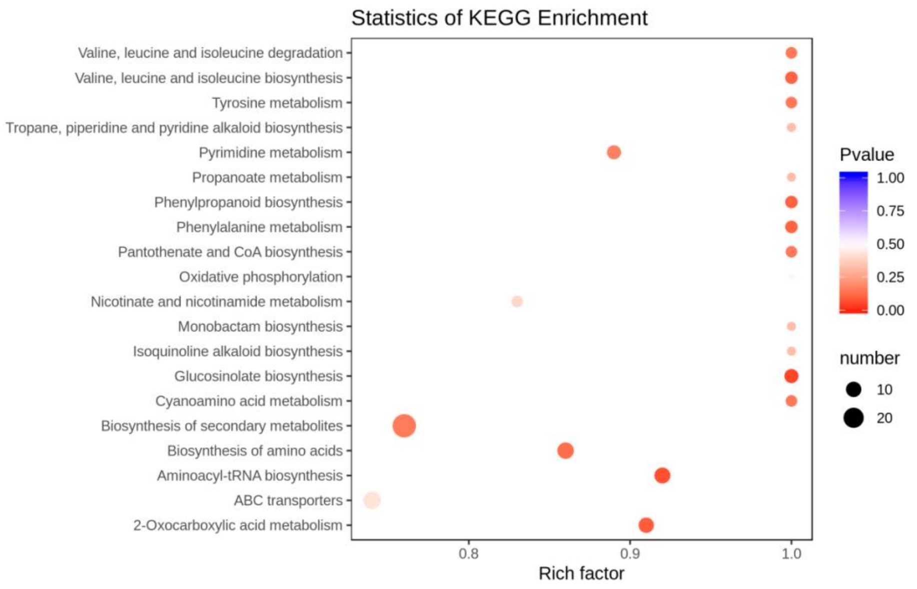

b

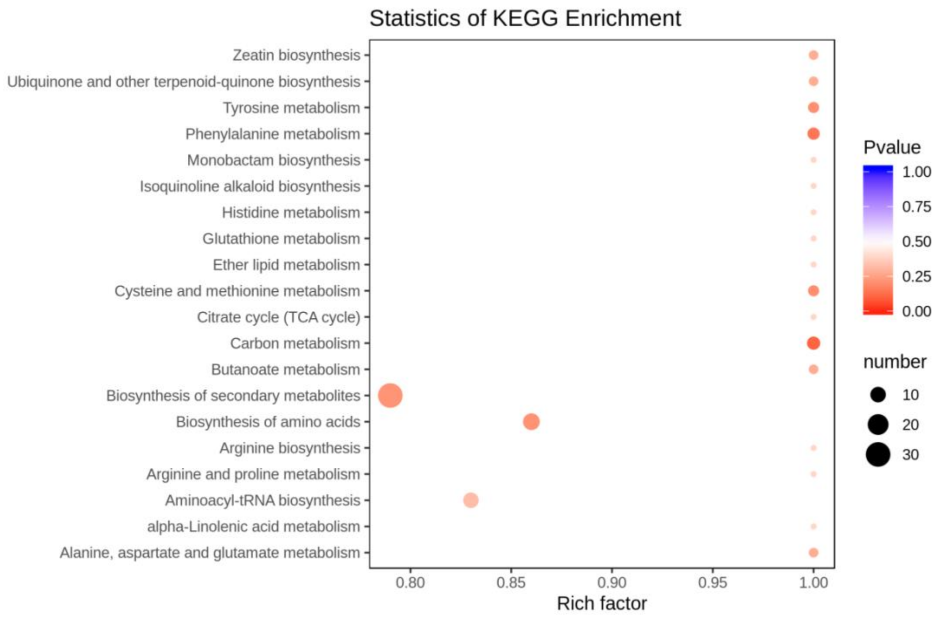

Figure S2. Enrichment analysis for KEGG pathways of DEMs between *A. correntina* and GH85. a: *A. correntina* -30d_vs_GH85-30d. b: *A. correntina* -60d _vs_GH85-60d.

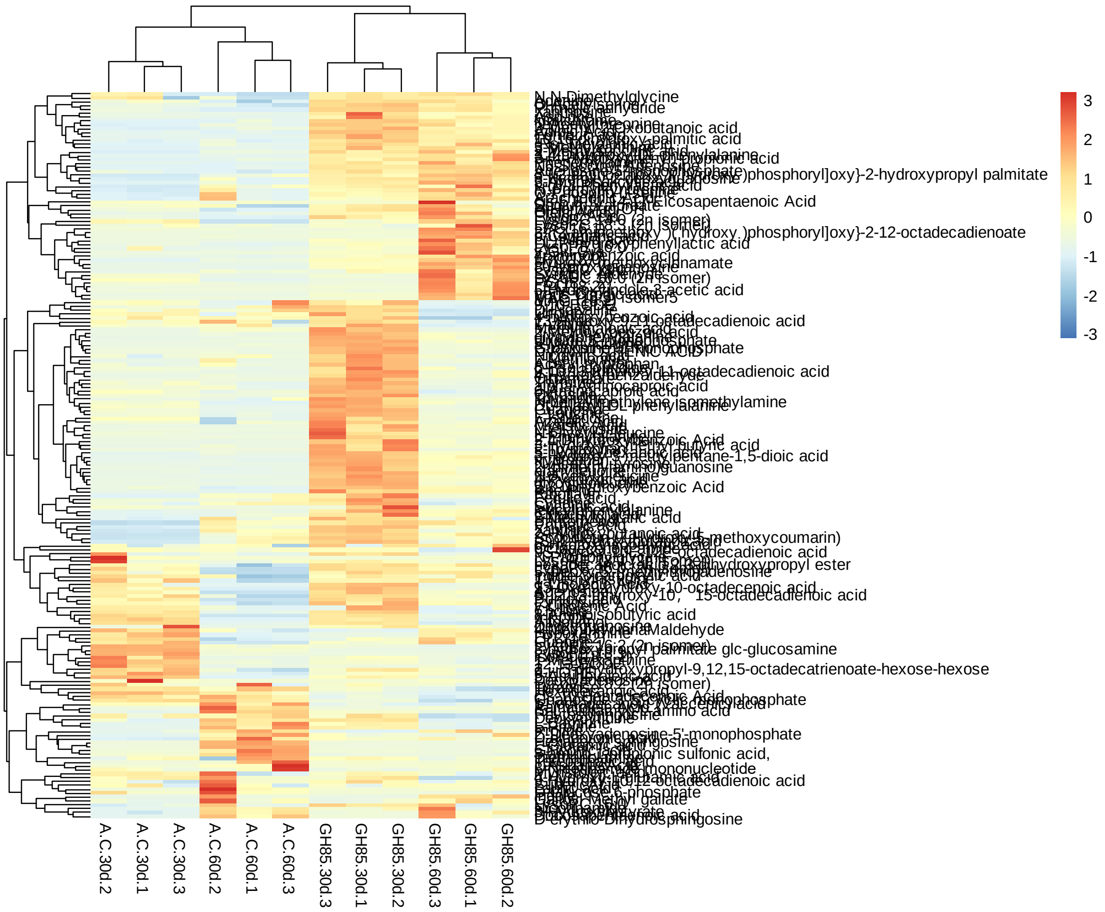

Figure S3. Clustering analysis diagram of DEMs between *A. correntina* and GH85
